# Anti-tumor necrosis factor agents and COVID-19: A word of caution

**Published:** 2020-08-29

**Authors:** Roy Hajjar, Gabriel Chan

**Affiliations:** ^1^Department of Surgery, Hôpital Maisonneuve-Rosemont, Montréal, Québec, Canada. 5415, boulevard de l’Assomption, Montréal, Québec, Canada; ^2^Division of General Surgery, Université de Montréal, Montréal, Québec, Canada

**Keywords:** COVID-19, tumor necrosis factor, anti-TNF

Anti-tumor necrosis factor-alpha (anti-TNF-α) agents have been suggested as being favorable [1] and a potential treatment for coronavirus disease of 2019 (COVID-19) [2]. High serum levels of TNF-α upon admission have been associated with a severe COVID-19 infection [3]. From the literature studying sepsis, non-related to COVID-19, a previous meta-analysis had found an improved survival, in particular 28-day all-cause mortality, with monoclonal anti-TNF antibodies [4]. In trials of acute inflammation, such as steroid-refractory and severe ulcerative colitis (UC), anti-TNF agents were shown to have a favorable adverse event profile, in addition to inducing higher remission rates and improving the percentage of colon-salvage [5-7].

The experience of IBD patients infected with COVID-19 has been reported through the SECURE-IBD database. As of July 13, 2020, 15% of the patients on anti-TNF therapy required hospitalization, with 2% requiring intensive care and 1% succumbing to the disease [8]. Patients treated with steroids or sulfasalazine/mesalamine required intensive care in 15% and 8% of the cases, respectively, and had a mortality of 9% and 6% [8]. It appears that in the setting of maintenance therapy with anti-TNF antibodies, COVID-19 infections are associated with less serious complications as compared to other maintenance modalities. How an anti-TNF-α therapy could work for patients who had not been on treatment at the onset of their COVID-19 infection has yet to be defined.

Furthermore, important differences exist between the study populations of IBD trials and COVID-19 patients. IBD trials typically exclude patients with severe uncontrolled infections and those with heart failure [6]. Severe COVID-19 infections would have likely been excluded from participation in these trials. Furthermore, the age group studied in these IBD trials tends to be in the 20-40-year-old range [7], much younger than the critically ill patients with a severe COVID-19 infection [9]. Although graded as severe, the disease burden of UC in such studies is usually confined to the intestine, without the outright systemic sepsis seen in severe COVID-19 cases.

The cumulative evidence in the literature has also been inconsistent regarding the safety profile for infectious adverse events with anti-TNF-α therapies in IBD. When compared to placebo, meta-analysis has found that UC patients treated with anti-TNF agents had increased long-term risks of opportunistic and of serious infections requiring intravenous antibiotics or hospitalization, or causing death [10]. A higher risk of serious infectious complications has also been shown in patients with autoimmune arthritis receiving anti-TNF-α agents [11]. In older patients with a severe COVID-19 infection, the safety profile leaves even less margin for error. Some reports have suggested a tolerable adverse event profile in the elderly with inflammatory bowel and articular diseases [12-14], while others have found a higher rate of opportunistic infections and treatment failure with IBD cases [15,16].

The indication of anti-TNF-α treatment in the present pandemic would target pulmonary inflammation and mortality. The previous literature concerning both acute lung injury and acute respiratory distress syndrome feature higher local and systemic levels of TNF-α and have been associated with worse outcomes [17]. Similar to the current COVID-19 pandemic, this suggests a possible role of TNF inhibitors in the treatment of acute pulmonary inflammation. However, previous reports have not shown favorable effects on the survival of TNF blockade in patients with septic shock and ALI [17,18].

It is nonetheless worth noting that emerging data on the use of anti-TNF agents in patients with COVID-19 included the description of a case where infliximab was initiated in a 54-year-old female patient for a severe episode of ulcerative colitis [19]. She specifically received two doses of infliximab 10 mg/kg, while being reported as having a concomitant infection with COVID-19 that was qualified as mild [19]. The patient had an uneventful recovery [19]. In their cohort of 40 patients with IBD, the authors reported two deaths (5%) due to acute respiratory distress syndrome in patients aged 77 and 86 years, and who were treated with mesalamine with or without methotrexate [19]. A complementary element to note with the reported fatality rate is the fact that most patients were described to have interrupted their immunomodulator or biologic maintenance therapy upon the diagnosis of a COVID-19 infection [19]. This adds an additional challenge to the proper interpretation of the safety of anti-TNF use during an active episode of COVID-19 and its potential effect on complications’ occurrence. Although the initiation of an anti-TNF agent in a patient with a mild case of COVID-19 seems safe and potentially beneficial, it remains important to note that more cases are required to properly assess which patients might benefit from its use as a therapeutic agent for COVID-19.

With no experience with this novel virus, TNF blockade, although mechanistically promising, may harbor serious risks in fragile older patients. We endorse the hypothesis stipulating that this therapy might prevent severe complications of systemic inflammation in COVID-19 infections, but further investigation into the evolution of COVID-19 infections in IBD patients already treated with anti-TNF agents could provide valuable insight into the benefits of suppressing the TNF-α cascade. Caution should, however, be used in projecting any outcomes observed in patients on maintenance therapy to initiating antibody treatment in a naïve but COVID-19 afflicted patients.
